# Evolutionarily Selected Overexpression of the Cytokine BAFF Enhances Mucosal Immune Response Against *P. falciparum*

**DOI:** 10.3389/fimmu.2020.575103

**Published:** 2020-10-06

**Authors:** Valeria Lodde, Matteo Floris, Isabel Beerman, Rachel Munk, Rajan Guha, Maristella Steri, Valeria Orrù, Kotb Abdelmohsen, Peter D. Crompton, Myriam Gorospe, Maria Laura Idda, Francesco Cucca

**Affiliations:** ^1^Department of Biomedical Sciences, University of Sassari, Sassari, Italy; ^2^Istituto di Ricerca Genetica e Biomedica, Consiglio Nazionale delle Ricerche (CNR), Monserrato, Italy; ^3^Epigenetics and Stem Cell Unit, Translational Gerontology Branch, National Institute on Aging, National Institutes of Health, Baltimore, MD, United States; ^4^Laboratory of Genetics and Genomics, National Institute on Aging Intramural Research Program, National Institutes of Health, Baltimore, MD, United States; ^5^Malaria Infection Biology and Immunity Section, Laboratory of Immunogenetics, National Institute of Allergy and Infectious Diseases, National Institutes of Health, Rockville, MD, United States

**Keywords:** malaria, BAFF, gene regulation, evolution, immune regulation

## Abstract

We have previously shown that a variant of the *TNFSF13B* gene that we called *BAFF-var* increases the production of the cytokine BAFF, upregulating humoral immunity and increasing the risk for certain autoimmune diseases. In addition, genetic population signatures revealed that *BAFF-var* was evolutionarily advantageous, most likely by increasing resistance to malaria infection, which is a prime candidate for selective pressure. To evaluate whether the increased soluble BAFF (sBAFF) production confers protection, we experimentally assessed the role of *BAFF-var* in response to malaria antigens. Lysates of erythrocytes infected with *Plasmodium falciparum* (iRBCs) or left uninfected (uRBCs, control) were used to treat peripheral blood mononuclear cells (PBMCs) with distinct BAFF genotypes. The PBMCs purified from *BAFF-var* donors and treated with iRBCs showed different levels of specific cells, immunoglobulins, and cytokines as compared with *BAFF-WT.* In particular, a relevant differential effect on mucosal immunity B subpopulations have been observed. These findings point to specific immune cells and molecules through which the evolutionary selected *BAFF-var* may have improved fitness during *P. falciparum* infection.

## Introduction

Malaria is one of the most prevalent infectious diseases and is also a global public health challenge. Several therapeutic strategies against malaria have been employed with considerable success, but a highly definitive vaccine remains elusive, partially due to an incomplete understanding of the immune response to *Plasmodium falciparum*, the most deadly malaria parasite species that infects humans (1–3).

The immunological response to malaria infection in humans is cooperatively regulated by both the innate and adaptive immune systems (4). Considerable evidence revealed that B cells, T cells, antibodies, cytokines, and their respective receptors all play crucial roles in the recruitment and activation of different cell types of the immune system, thus modulating the complex immunological response against malaria parasites (2, 3). B cells are essential for long-term maintenance of a protective humoral immunity to *P. falciparum*, and antibodies are a major component of the immune response during the erythrocytic stages (5). T lymphocytes play two key roles in malaria immunity: (i) they act as helper cells in the production of anti-*plasmodium* immunoglobulin, and (ii) they trigger cell-mediated mechanisms that activated macrophages and other phagocytic or cytotoxic cells (6). Additionally, physical interaction between B cells and CD4+ helper T cells induces the secretion of cytokines that contribute to the immune modulation of the anti-malaria response. Several studies underscore the importance of the cytokine balance in immune protection against malaria (7, 8). Upregulation of pro-inflammatory cytokines such as IFN-γ (IFNG), IL12, and TNF-α (TNF) during the early stages of infection contributes to protection and resolution of parasite infection (9). However, an appropriately balanced release of pro- and anti-inflammatory cytokines is critical for a positive outcome of malaria disease. Indeed, defects in the production of TGF-β (TGFB1) and IL10, two anti-inflammatory factors, are associated with acute, severe malaria, severe malaria anemia, and an overall negative outcome (10–12).

We recently identified a genetic variant (var) in the *TNFSF13B* gene encoding the cytokine, B-cell-activating factor (BAFF), which has long been known to be critical for the proliferation, differentiation, and survival of B cells. The new variant (var) arises from an insertion/deletion (GCTGT > A) that introduces an alternative polyadenylation (APA) site in the 3′-untranslated region (UTR) of *BAFF* mRNA. The presence of the APA causes the production of a shorter transcript (*BAFF-var* mRNA) that is more actively translated than the longer, wild-type transcript (*BAFF-WT* mRNA) (13). The RNA-binding protein (RBP) NF90 and the microRNA miR-15a cooperatively reduce BAFF production from *BAFF-WT* mRNA (13, 14). Accordingly, donors carrying the *BAFF-var* allele have a higher level of soluble BAFF (sBAFF) in their serum and an increased risk of developing autoimmunity (13). The presence of *BAFF-var* was also significantly associated with several immune-related diseases, increased number of circulating B lymphocytes and serum immunoglobulins (Ig) M, A, and G, and a decreased number of monocytes (13).

Population genetic studies have found evidence of strong positive selection for *BAFF-var* (13), possibly due to the adaptive advantage it confers against infectious diseases. Indeed, *BAFF-var* had a higher frequency in Sardinia compared to mainland Italy and other regions worldwide and showed a progressive reduction in frequency from the Mediterranean area to Northern Europe (13). Interestingly, *BAFF-var* is absent or extremely rare in Africa and Asia, suggesting that it originated after the out-of-Africa movement of modern humans and became common in areas endemic for malaria such as Sardinia (13). Therefore, we have assessed whether the high frequency of *BAFF-var* in Sardinia was consistent with the effects of random genetic drift or was instead a consequence of positive selection, favoring *BAFF-var* particularly in Sardinia. Haplotype-based selection analyses showed that the core haplotype carrying *BAFF-var* was remarkably larger than haplotypes carrying variants with matched genetic features. This finding and the high allelic frequency were consistent with the hypothesis of positive selection acting on *BAFF-var* (13). Due to its effects on humoral immunity, the *BAFF-var* allele may have been selected for improved fitness against infections such as malaria, which was highly prevalent in Sardinia until its eradication in the early 1950s (15). This hypothesis was further supported by a report that mice overexpressing human sBAFF were protected against lethal *Plasmodium yoelii* infection, thereby confirming the key role that BAFF plays during malaria infections (16).

To identify the possible cellular and molecular mechanisms underlying the protection against malaria exerted by the inherited *BAFF-var* allele, we set up appropriate *in vitro* assays. Lysates of erythrocytes infected with *P. falciparum* (iRBCs) or left uninfected (uRBCs, control), were used to treat peripheral blood mononuclear cells (PBMCs) with the *BAFF-var* genotype. PBMCs purified from *BAFF-var* donors and treated with iRBCs showed different levels of B- and T-cell traits [subpopulations and mean fluorescence intensity (MFI)], as well as differential expression of immunoglobulins, cytokines, and other genes as compared with the levels observed in PBMCs from *BAFF-WT* donors. In particular, a relevant differential effect on β7 integrin in B subpopulations was observed. Furthermore, RNA sequencing of total B cells, purified from PBMCs treated with iRBCs or uRBCs, identified several transcripts differentially expressed, including some that encoded proteins critical for the response to malaria infection, CXCL10, CR1, ICAM1, MIF, and NFkB2. *In vivo* testing on the role of BAFF-var in mitigating the clinical picture of malaria in affected individuals is not possible because this variant is common in Sardinia, where malaria was eradicated about 70 years ago, while it is absent in areas of the world where the infection is still widespread.

Taken together, our findings identified a previously unknown mechanism by which expression of the *BAFF-var* allele increases sBAFF levels in humans and potentiates the immune system against *Plasmodium* infection, which concurrently increased the risk of autoimmune diseases. We propose that these results will help with the rational design of BAFF-based therapies in the treatment of malaria infection.

## Materials and Methods

### Preparation of *P. falciparum*-Infected Red Blood Cell (RBC) Lysates

*Plasmodium falciparum*-infected RBC lysates were prepared as previously described (17). Briefly, 3D7 *P. falciparum* cultures were maintained in fresh human O^Rh+^ erythrocytes at 3% hematocrit in RPMI 1640 medium (KD Medical) supplemented with 10% heat-inactivated O^Rh+^ human serum (Interstate Blood Bank, Memphis, TN, United States), 7.4% sodium bicarbonate (GIBCO, Invitrogen), and 25 μg/ml of gentamycin (GIBCO, Invitrogen), at 37°C in the presence of a gas mixture containing 5% O_2_, 5% CO_2_, and 90% N_2_. Mycoplasma-free cultures of *P. falciparum* schizont-infected red blood cells (iRBCs) were isolated using magnetic columns (LD MACS Separation Columns, Miltenyi Biotec). Lysates of red blood cells infected (iRBCs) and uninfected (uRBCs) with *P. falciparum* were obtained by three cycles of freeze–thaw, liquid nitrogen followed by 37°C in water bath. The infection was conducted by treating human cells with a specific number of RBC lysates, infected and uninfected with *P. falciparum*. This number is indicated as cell:lysate ratio.

### Cell Cultures

Primary PBMCs were purified using Histopaque^®^-1077 (Sigma) from genotyped healthy human donors of the SardiNIA general population cohort (13) and homozygotes for *BAFF-WT* (*n* = 10) and for *BAFF-var* (*n* = 11) (Table 1). Cells were stored in liquid nitrogen and then thawed and cultured in RPMI 1640 medium supplemented with 10% fetal bovine serum and 1% Pen/Strep in a 37°C, 5% CO_2_ incubator. PBMCs were treated for 48 h with iRBCs or uRBCs using 1:3 ratio (PBMC:lysate).

**TABLE 1:** Sex, age, genotype, and sBAFF production (pg/ml) of the donors included in the study.

**Donor n.**	**Sex**	**Age**	**Genotype**	**Serum sBAFF (pg/ml)**
1	M	83	BAFF-var	1,444,631
2	M	75	BAFF-WT	494,872
3	F	61	BAFF-WT	616,274
4	M	80	BAFF-WT	711,395
5	M	76	BAFF-var	893,585
6	M	78	BAFF-var	1,009,934
7	F	74	BAFF-var	1,115,324
8	M	80	BAFF-WT	557,831
9	F	61	BAFF-var	1,220,857
10	F	40	BAFF-var	1,182,851
11	F	50	BAFF-var	887,712
12	F	51	BAFF-var	885,86
13	M	52	BAFF-var	1,289,649
14	M	46	BAFF-WT	497,322
15	M	72	BAFF-var	885,532
16	F	51	BAFF-WT	516,019
17	M	56	BAFF-WT	588,818
18	M	64	BAFF-var	852,21
19	M	69	BAFF-WT	598,337
20	M	58	BAFF-WT	543,334
21	F	54	BAFF-WT	454,729

The incubation time and concentration of lysates were determined experimentally previously by treating only PBMC-WT with different concentration of lysates and at different time points (data not shown). The condition leading to the highest sBAFF production was used in all the experiment presented in the manuscript.

### B Cell Isolation

Primary B cells were isolated from PBMCs treated with lysate of *P. falciparum* (iRBCs and uRBCs). After the 48-h treatment, B cells were purified using the Easy Sep negative selection system (STEMCELL Technologies). Briefly, treated PBMCs were resuspended in PBS containing 2% fetal bovine serum and 1 mM EDTA and Enrichment Cocktail, containing the antibody complex. After incubation for 10 min, 50 μl of magnetic beads and recommended media were added. The samples were then placed into the magnetic support for 5 min to isolate the B cells.

### FACS Analysis

Peripheral blood mononuclear cells were washed in PBS with 1% BSA (bovine serum albumin) and incubated for 30 min at 25°C with fluorescently labeled antibodies specific for B and T cells. Subsequently, samples were centrifuged and resuspended in propidium iodide (PI) solution (1 μg/ml PI and 10 μg/ml RNase A in PBS) and analyzed using BD FACS Canto II flow cytometer (BD Biosciences). The B cell panel included the following antibodies: IgA FITC, IgD PE, CD3 PerCP-Cy5.5, CD27 APC-H7, CD19 PE-Cy7, β7 APC, and CD38 BV421 from BD Biosciences. The T cell panel included the following antibodies: CXCR5 BV421, CXCR3 PE, CD4 APC-H7, CD3 FITC, CD196 APC, CD279 (PD-1) BV510, and CD45RA PE-Cy7 from BD Biosciences. Results were analyzed using FACSDiva software (BD Biosciences) and reported as MFI, reflecting the levels of cell surface antigens and relative cell count with respect to hierarchically higher cell populations (%). Cellular aggregates were eliminated using morphology parameters (FSC-A and FSC-H) (see Supplementary Figures 1, 2 for gating strategy).

### ELISA

Relative soluble protein levels were measured in collected supernatants of PBMCs (*BAFF-WT* or *BAFF-var)* by enzyme-linked immunosorbent assay (ELISA) and by Bio-Plex Multiplex Immunoassay System (Bio-Rad Laboratories). BAFF protein levels were measured using an ELISA Kit (AdipoGen). The plate was analyzed at 450 nm with a SUNRISE TECAN plate reader. All the other cytokines and immunoglobulins were measured using a Bio-Rad Bio-Plex plate: for human cytokine analysis, we used the Pro Human Cytokine 17-plex assay, and for immunoglobulin analysis, we used the human IgG total isotyping assay according to the manufacturer’s instructions (Bio-Rad Laboratories). Finally, the plates were read with Bio-Plex 200 instrument (Bio-Rad).

### RNA Isolation, Reverse Transcription (RT)-Quantitative (q)PCR Analysis

RNA was isolated from PBMCs and B cells using the TriPure isolation reagent (Roche) following the manufacturer’s protocol. Total RNA was reverse-transcribed (RT) into cDNA using Maxima reverse transcriptase (Thermo Fisher Scientific) and random hexamers. The resulting cDNA was analyzed by quantitative (q) PCR using SYBR Green mix (Kapa Biosystems). The relative levels of RNAs were calculated by the 2^–Δ^ ^*Ct*^ method and β*-actin* (*ACTB*) mRNA levels were used for normalization. The gene-specific primers used are listed in Table 2.

**TABLE 2:** List of primers used for qPCR.

**RT-(q)PCR primers**	**Sequences (5′–3′)**
*CXCL10*	FW: AAACTGCCATTCTGATTTGCT
	RV: TTGAATGCCACTTAGAGTCAA
*NFKB2*	FW: GATCGAGGTGGACCTGGTAA
	RV: GGGCAGTCATGTCCTTGG
*MIF*	FW: TCAACTATTACGACATGAACGCG
	RV: CTTAGGCGAAGGTGGAGTTG
*CR1*	FW: CCCATTGGGACATATCTGAAC
	RV: GCACCAGTCCAGACTGAGTTTT
*ICAM1*	FW: TGTCCCCCTCAAAAGTCATC
	RV: GGGTCTCTATGCCCAACAAC
*IL8*	FW: GAGTGGACCACACTGCGCCA
	RV: TCCACAACCCTCTGCACCCAGT
*IFN*γ	FW: TTTTCAGCTCTGCATCGTTTT
	RV: TCCGCTACATCTGAATGACCT
*MIP-1B*	FW: ACCGCCTGCTGCTTTTCTTA
	RV: CAGAGGCTGCTGGTCTCATA
*GM-CSF*	FW: CTGCTGCTCTTGGGCACT
	RV: GGATGGCATTCACATGCTC
*IL10*	FW: GGCACCCAGTCTGAGAACAG
	RV: CTTCACTCTGCTGAAGGCATC
*MYC*	FW:TGGGAGGAGACATGGTGAAC
	RV:TTCTCTGAGACGAGCTTGGC
*GAPDH*	FW:TGCACCACCAACTGCTTAGC
	RV:GGCATGGACTGTGGTCATGAG
18s	FW:GGAGAGGGAGCCTGAGAAAC
	RV:TCGGGAGTGGGTAATTTGC
*ACTB*	FW: CATGTACGTTGCTATCCAGGC
	RV: CTCCTTAATGTCACGCACGAT

### RNA Sequencing

Total RNA from B cells, isolated from PBMCs after treatment with iRBCs and uRBCs for 48 h, was sequenced and libraries were prepared according to SMARTer Stranded Total RNA-Seq Kit v2 (Takara Bio). Paired-end sequencing was performed on an Illumina HiSeq 4000 instrument (Illumina Inc.). For the bioinformatic analysis of RNAseq data, we performed adapter trimming of fastq files with the tool TrimGalore v0.4.5 with parameters –“Illumina –paired –phred33 –clip_r1 3 –three_prime_clip_r2 3.” After trimming, we used STAR to align the paired-end reads to the human reference genome (build 38) with parameter “quantMode GeneCounts.” We then used Picard Tools to de-duplicate and sort the alignments. The feature-Count tools were then used to count how many reads have mapped to each human gene (using the Gencode v29 basic annotation schema). The DESeq2 algorithm was then used to determine significant differences in expression (counts) between the different experimental conditions. Finally, data were compared with Phenopedia database^1^ using Venny^2^.

### Western Blot Analysis

Whole-cell lysates were prepared using RIPA buffer, size-fractionated through 4–12% gradient polyacrylamide gels (Thermo Fisher Scientific), and transferred to nitrocellulose membrane using Trans-Blot Turbo RTA Transfer Kit, Nitrocellulose (Bio-Rad). Membranes were incubated for 16 h with primary antibodies recognizing NFKB2 (Cell Signaling Technologies), CXCL10 (Abcam), MIF (Abcam), CR1 (Abcam), ICAM1 (Abcam), and ACTB (Santa Cruz Biotechnology), as described in Table 3. After incubation with the appropriate secondary antibodies, conjugated with horseradish peroxidase (HRP), the signals were detected by enhanced chemiluminescent reaction using SuperSignal West Femto (Thermo Fisher Scientific). Images were acquired with the Bio-Rad Universal Hood II Gel Doc System.

**TABLE 3:** List of antibodies and relative dilutions used for Western blot.

**Primary antibody**	**Dilution**
NFKB2	1:500
CXCL10	1:500
MIF	1:500
CR1	1:500
ICAM1	1:500
ACTB	1:1000

### Analysis of mRNA Stability by Actinomycin D Assays

For mRNA half-life determination, PBMCs (WT and variant) were treated for 48 h with iRBCs and uRBCs and then incubated with Actinomycin D (Act D; 5 μg/ml) to block *de novo* transcription. Cells were harvested at subsequent time intervals (0, 0.5, 1, 2, 4, and 6 h) of Act D treatment, and total RNA was extracted and processed as described above by RT-qPCR analysis, although mRNA levels were normalized to *18S* rRNA levels. Data from Act D assays were processed using the Prism 7 software to assess mRNA decay curves.

### Statistical Analysis

Statistical significance was determined using two-tailed *t*-tests, as indicated in the figure legends. Values were considered significant when *P* < 0.05. Tests of statistical significance were conducted using Prism 7 software (GraphPad).

## Results

### Autoimmunity Variant *BAFF-var* in Sardinians Enhanced sBAFF Production

**FIGURE 1:**
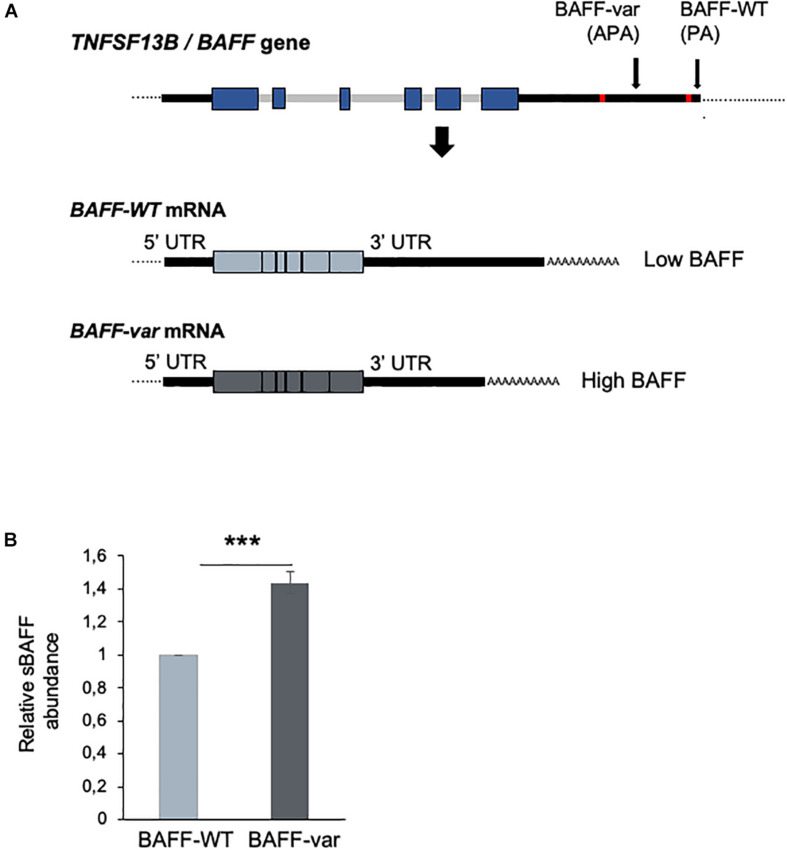
Differential expression of BAFF from cells with *BAFF-var* and *BAFF-WT* genotypes. **(A)** Top, schematic representation of the *TNFSF13B*/*BAFF* gene, polyadenylation site (PA), and alternative polyadenylation site (APA) generated in the presence of the *BAFF-var* allele. Bottom, mRNAs generated from the *TNFSF13B*/*BAFF* gene according to polyadenylation site usage [*BAFF-WT* mRNA (light blue) and *BAFF-var* mRNA (gray)]. **(B)** Relative soluble BAFF protein levels measured by ELISA from supernatants of cultured cells from individuals homozygous for *BAFF-WT* (*n* = 10) or for *BAFF-var* (*n* = 11). Cells were treated with uRBCs for 48 h. Data in **(B)** are the means and standard deviation (+SD) from at least three independent experiments. ***P < 0.005.

The insertion–deletion variant in the 3′UTR of the *TNFSF13B* gene (encoding BAFF) associated with increased risk of autoimmune diseases creates an APA site that generates a shorter transcript (*BAFF-var* mRNA) that is more efficiently translated and hence produces higher levels of sBAFF (13, 14) (Figure 1A). To investigate the mechanism through which *BAFF-var* may have been selected for improved fitness against malaria infections, we used lysates of erythrocytes infected with *P. falciparum* (iRBCs) or left uninfected (uRBCs, control) to treat primary cells from Sardinian donors with different BAFF genotypes. Initially, we confirmed that sBAFF production was higher in *BAFF-var* donors compared with *BAFF-WT* donors after *in vitro* culture. To this end, PBMCs from Sardinia donors were cultured with uRBCs for 48 h and sBAFF levels were measured from the conditioned media by ELISA. As observed, supernatants from PBMCs with both *BAFF-WT* alleles (PBMC-WT) had lower sBAFF levels than the supernatants from PBMCs with both *BAFF-var* alleles (PBMC-var) (*n* = 11) (Figure 1B), in agreement with our previous publication (13).

### Effect of Malaria Antigens on PBMC-WT and PBMC-var Populations

**FIGURE 2:**
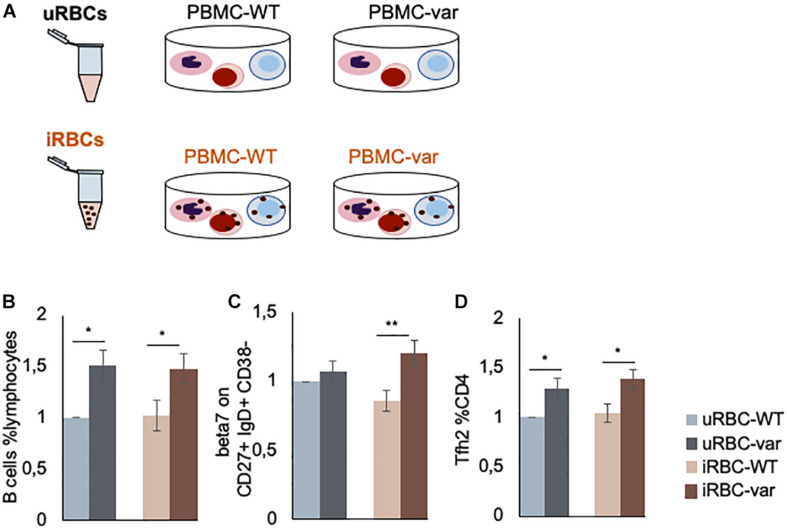
*P. falciparum* antigens influence the production of B and T cells. **(A)** Schematic representation of the work design. PBMCs expressing normal (*BAFF-WT*) or higher (*BAFF-var*) levels of sBAFF were treated for 48 h with lysates of red blood cells uninfected or infected with *P. falciparum* (uRBCs and iRBCs, respectively). **(B–D)** PBMC-WT (*n* = 10) and PBMC-var (*n* = 10) were treated for 48 h with uRBCs or iRBCs. Cells were stained to identify B and T cell subpopulations, treated with propidium iodide solution, and analyzed by fluorescence-activated cell sorting (FACS). **(B)** Relative number of B cells with respect to total lymphocytes (%). **(C)** Expression levels of integrin β7 on the indicated cell populations (MFI). **(D)** Relative number of T cell sub-populations with respect to T helper (CD4+) cells (%). Each panel shows the average of the relative expression for each population. Data are the means and standard deviation (+SD) from at least three independent experiments. *P < 0.05, **P < 0.01.

To test if the *BAFF-var* status enhances the immune response to malaria, PBMCs purified from *BAFF-WT* and *BAFF-var* donors were treated with lysates of uRBCs or iRBCs for 48 h. Four different conditions were compared: (i) PBMC-WT treated with uRBCs (uRBC-WT), (ii) PBMC-WT treated with iRBCs (iRBC-WT), (iii) PBMC-var treated with uRBCs (uRBC-var), and (iv) PBMC-var treated with iRBCs (iRBC-var) (Figure 2A). To identify any enrichments in subpopulations of B and T cells, the treated PBMCs were analyzed by flow cytometry (Figures 2B–D). PBMC-WT cells cultured with uRBCs were used as a control group to normalize the other conditions.

In line with our previous results obtained using samples from 2000 donors (13), we observed a higher percentage of B cells with respect to total lymphocytes in *BAFF-var* samples as compared to *BAFF-WT* in the absence of iRBC stimulation (Figure 2B). Interestingly, this trend is observed for both uninfected and infected samples supporting a role for the *BAFF-var* allele in expanding the B cell population, which is fundamental in *P. falciparum* immunity. Furthermore, as the involvement of mucosal immunity in *Plasmodium* infection was previously demonstrated (17, 18), assessment of β7 integrin cell surface marker expression levels (represented as MFI) on the B cell subsets was increased in *BAFF-var* samples following exposure to *Plasmodium* antigens. The β7 integrin MFI signals were particularly strong for CD27+ IgD+ CD38− cells, representing a subpopulation of unswitched memory B lymphocytes (Figure 2C). Finally, among T cells, the *BAFF-var* PBMCs displayed significant increases in the PD1+ CXCR5+ Th2 cell populations (Tfh2, Figure 2D), independent of the *P. falciparum* exposure.

### Effect of *P. falciparum* Antigens on Production of Immunoglobulins and Cytokines

**FIGURE 3:**
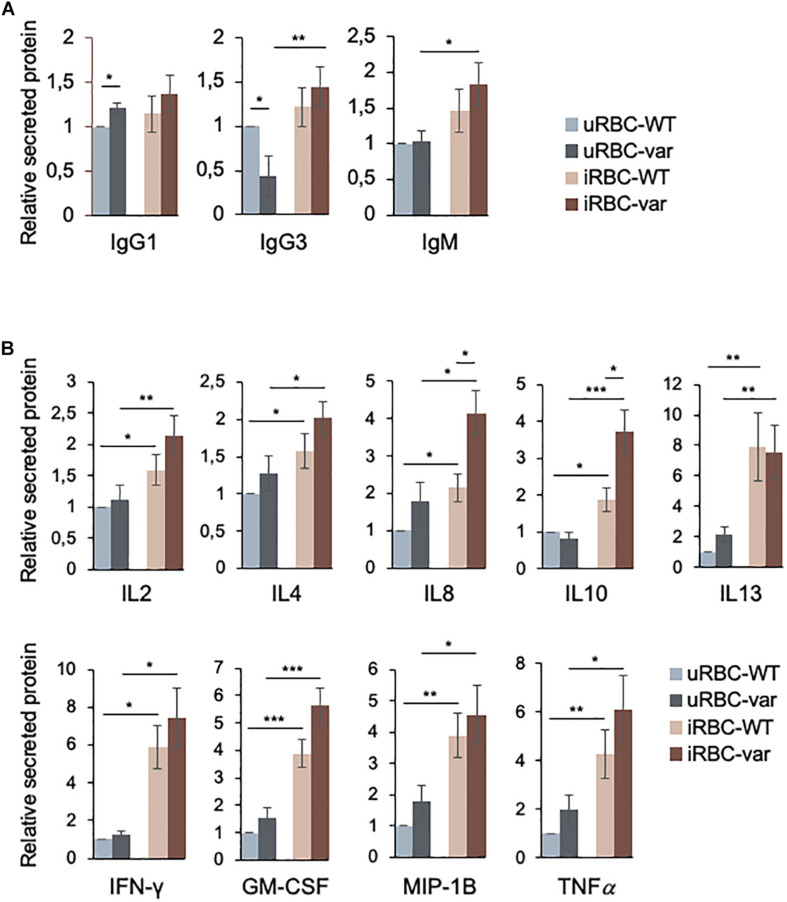
*BAFF-var* and *P. falciparum* antigens modulate the production of immunoglobulins and cytokines. PBMC-WT and PBMC-var were treated for 48 h with uRBCs or iRBCs. Supernatants were collected and the relative levels of soluble immunoglobulins **(A)** and cytokines **(B)** were quantified by Bio-Plex assay as described in section “Materials and Methods.” Data are the means and standard deviation (+SD) from at least three independent experiments. *P < 0.05, **P < 0.01, ***P < 0.005.

Antibodies are known to play a key role in naturally acquired immunity to the blood stage of *P. falciparum* infection. Until a few years ago, most studies focused on understanding the role of IgG in the response to *P. falciparum* infection, with IgG1 and IgG3 considered to be the most protective (19, 20); however, a role of IgM in malaria infection has also been considered more recently (21). We analyzed the presence of soluble Ig proteins using the Bio-Plex multiplex (Bio-Rad) system. As shown, IgG1 and IgG3 were differentially secreted by PBMCs exposed to uninfected lysates (uRBCs) as a function of BAFF status (Figure 3A), while exposure to *P. falciparum* antigens (iRBCs) significantly increased the production of IgG3 and IgM (in PBMC-var cells). Other immunoglobulins analyzed in the multiplex panel did not show significant differences (data not shown).

The balance between pro- and anti-inflammatory cytokines plays a crucial role in antimalarial immunity. Thus, the secretion of cytokines produced by PBMC-WT and PBMC-var was assessed using the Bio-Plex multiplex system (see section “Materials and Methods”). While for all the cytokines shown there was differential production depending on the presence of *P. falciparum* antigens (Figure 3B), only IL8 and IL10 were found to be differentially produced due to *BAFF-var* status.

**FIGURE 4:**
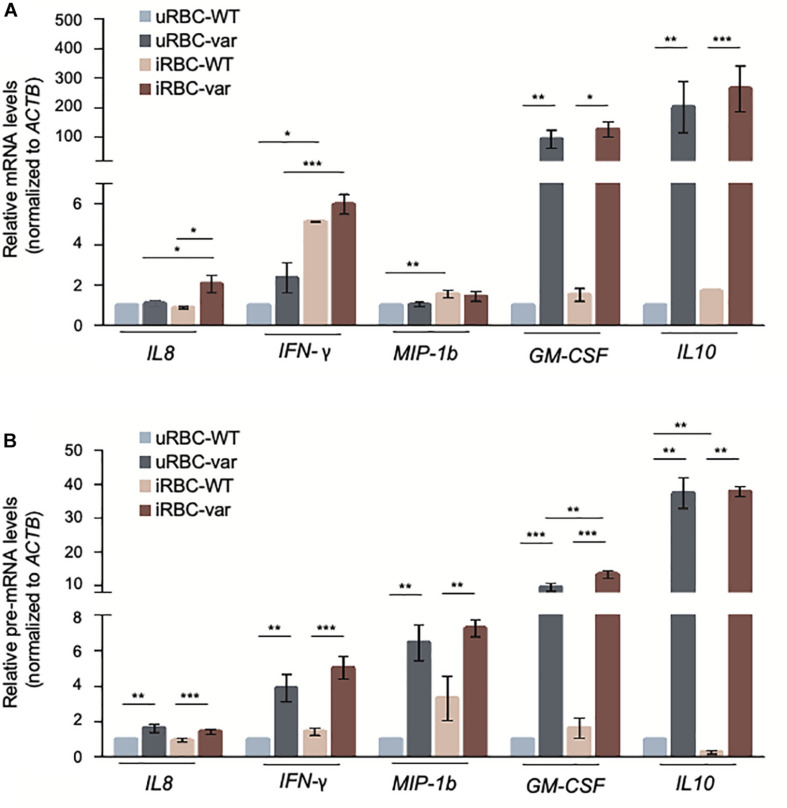
*P. falciparum* antigens activate cytokine expression in PBMCs. PBMC-WT and PBMC-var were treated as described in Figure 3; cell pellets were collected and RNA purified. The levels of the indicated mRNAs **(A)** and pre-mRNAs **(B)** were measured by RT-qPCR analysis. mRNA and pre-mRNA levels were normalized to *ACTB* mRNA levels. Data in **(A,B)** are the means and standard deviation (+SD) from at least three independent experiments. *P < 0.05, **P < 0.01, ***P < 0.005.

We then assessed if the changes in cytokine production by PBMCs were due to altered levels of the encoding mRNAs and pre-mRNAs. As shown (Figure 4), the levels of *GM-CSF* and *IL10* pre-mRNA and mRNA were strongly influenced by the presence of *BAFF-var*, suggesting a robust transcriptional regulation of these genes. All the other transcripts showed significant but modest changes: *IL8* mRNA levels increased in PBMC-var exposed to malaria antigens, and *IFN-*γ and *MIP-1b* mRNAs increased in the presence of *P. falciparum* antigens regardless of BAFF status (Figure 4).

### *P. falciparum* Antigens Altered the Transcriptome of B Cells in a *BAFF-var*-Dependent Manner

**FIGURE 5:**
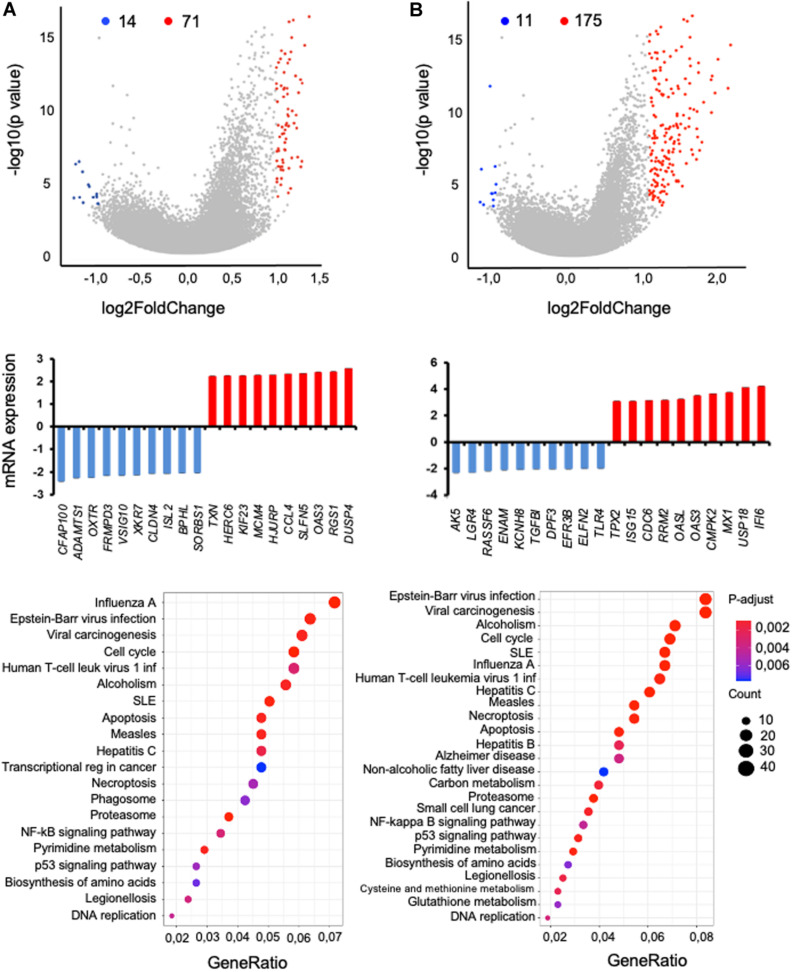
RNA-seq analysis of B cells isolated from PBMCs (WT or var) exposed to *P. falciparum* antigens. Two comparisons were studied: **(A)** iRBC-WT versus uRBC-WT and **(B)** iRBC-var versus uRBC-var. (Top) Volcano plot representation of the differential abundance of RNAs in B cells in the two groups; reduced mRNAs (blue) and increased mRNAs (red) are highlighted. (Middle) Among the mRNAs showing differential abundance, those displaying the greatest fold increases (red) and decreases (blue) by RNA-seq analysis were plotted. (Bottom) KEGG pathway enrichment analysis of the mRNAs differentially expressed in B cells from volcano plot.

In malaria, humoral immunity is important in disease outcome, yet to escape the host immune responses, *Plasmodium* parasites may specifically disturb the functionality of B cell subsets. Furthermore, as mentioned above, we previously observed a strong association of BAFF-var with increased circulating B cells in Sardinian donors (13). Thus, we assessed the global impact of *BAFF-var* on malaria infection by analyzing total RNA expression patterns in B cells. We treated PBMC-WT and PBMC-var with uRBCs or iRBCs for 48 h, and then isolated B cells as described in Section “Materials and Methods.” RNA was extracted for each condition and analyzed by RNA sequencing (RNA-seq), and gene expression patterns were compared as follows. First, B cells (WT) treated with iRBCs compared with B cells (WT) treated with uRBCs (Figure 5A); second, B cells (var) treated with iRBCs compared with B cells (var) treated with uRBCs (Figure 5B). RNA-seq analysis identified 71 genes upregulated (red dots) and 14 genes downregulated (blue dots) when comparing iRBC-WT with uRBC-WT (Figure 5A, top) and 175 genes upregulated and 11 genes downregulated when comparing iRBC-var with uRBC-var, *P* < 0.05 (Figure 5B, top and Supplementary Data set S1).

For the RNAs most differentially expressed, relative levels were calculated (Figures 5A,B, middle). Quantification of these signals revealed that in iRBC-WT versus uRBC-WT comparisons, *ADAMTS1*, *FRMPD3*, and *ISL2* mRNAs were downregulated, while *CCL4*, *RGS1*, and *OAS3* mRNAs were upregulated (Figure 5A, middle). Comparing the iRBC-var group with the uRBC-var group, *TLR4* and *TGFBI* mRNAs were downregulated, while *CDC6, MX1*, and *IFI6* mRNAs were upregulated (Figure 5B, middle). KEGG pathway analysis of the mRNAs differentially regulated in each dataset revealed that the encoded proteins are implicated in biological processes related to infectious diseases, autoimmunity, and the NFkB pathway, consistent with our previous results (Figures 5A,B, bottom).

### *BAFF-var* Modulated the Response to *P. falciparum* Antigens

**FIGURE 6:**
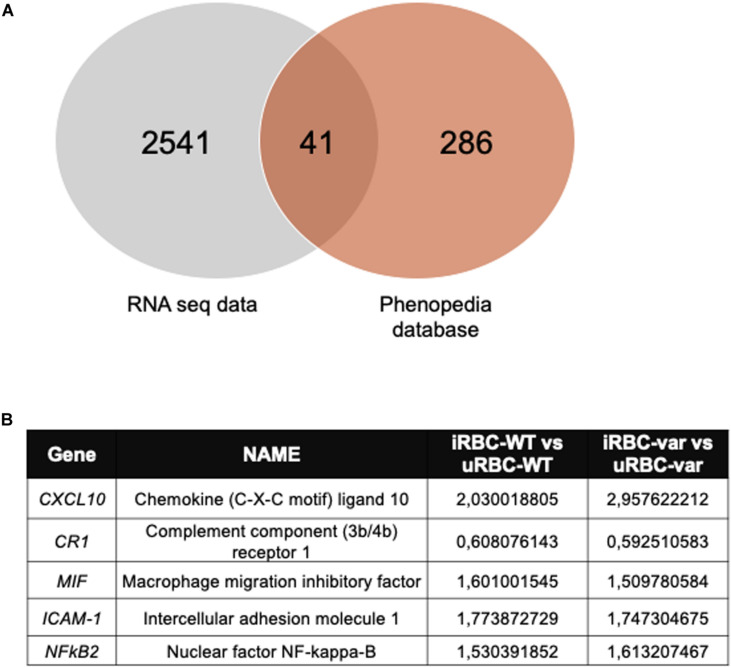
Selected mRNAs encode proteins implicated in the immune response to malaria infection. **(A)** Venn diagram illustrating the number of overlapping genes identified in the RNA seq data and malaria-related genes from the Phenopedia database. **(B)** Selected genes implicated in the immune response to malaria infection; fold changes in iRBC-WT versus uRBC-WT and iRBC-var versus uRBC-var are indicated.

**FIGURE 7:**
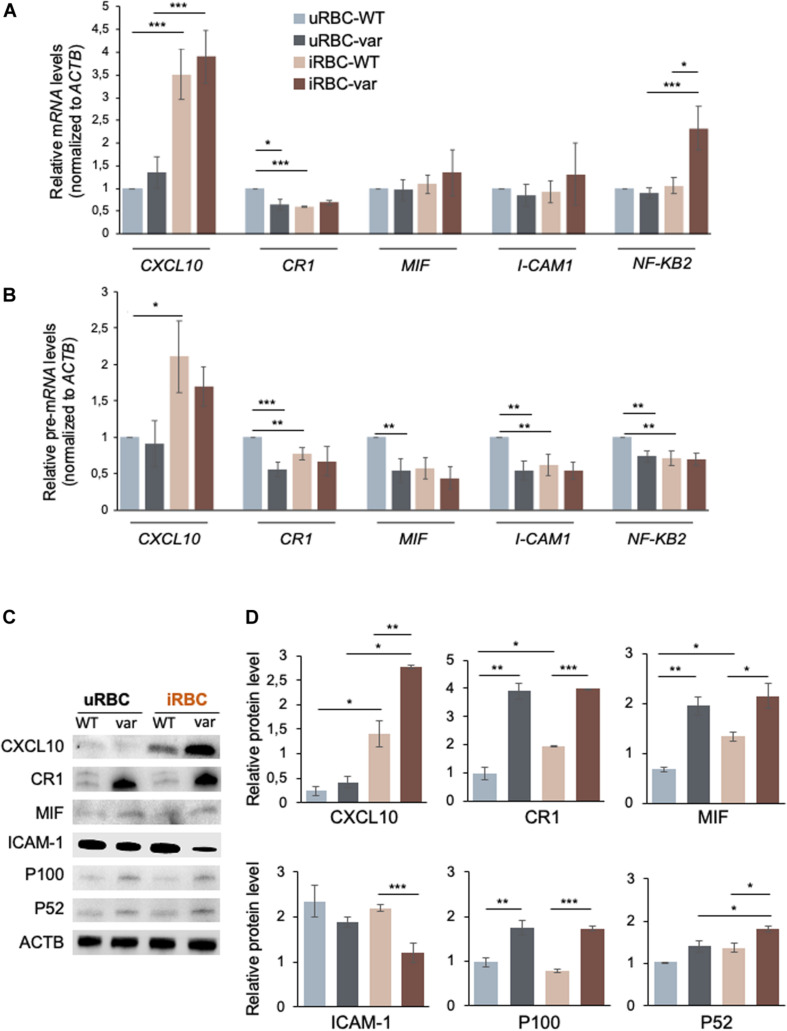
*BAFF-var* modulates the expression of pre-mRNAs, mRNAs, and proteins expressed in response to exposure to *P. falciparum* antigens. Protein and RNA purified from B cells isolated from PBMCs treated for 48 h with iRBCs and uRBCs were analyzed by Western blot and RT-qPCR, respectively. **(A,B)** Total RNA was purified for each condition and used for RT-qPCR to examine the expression of the indicated mRNAs **(A)** and pre-mRNAs **(B)**; mRNA and pre-mRNA levels were normalized to *ACTB* mRNA. **(C,D)** Protein expression levels were assessed by Western blot analysis **(C)** and protein signals were quantified using ImageJ and plotted **(D)**. β-actin (ACTB) served as a loading control. Data in **(A,B,D)** are the means and standard deviation (+SD) from at least three independent experiments. *P < 0.05, **P < 0.01, ***P < 0.005.

By intersecting the significantly modulated genes identified by RNA sequencing with genes directly involved in antimalarial immunity using the Phenopedia database (see text footnote 1) (22), we identified 41 candidate genes (Figure 6A) from which we selected a small group for further analysis: *CXCL10*, *CR1*, *I-CAM1*, *MIF*, and *NFKB2* (Figure 6B). The levels of the corresponding mRNAs and pre-mRNAs were measured by RT-qPCR analysis. *CXCL10* mRNA was upregulated in samples exposed to *P. falciparum* antigens (iRBC-WT and iRBC-var compared to uRBC-WT and uRBC-var); however, this increase significantly exceeded the observed rise in *CXCL10* pre-mRNA levels, suggesting that *CXCL10* mRNAs may be post-transcriptionally stabilized (Figures 7A,B). CXCL10 protein levels reflected *CXCL10* mRNA levels (Figures 7C,D). *CR1* pre-mRNA and mRNA changed moderately but significantly in iRBC-WT versus uRBC-WT and uRBC-var versus uRBC-WT (Figures 7A,B). Interestingly, CR1 protein levels changed dramatically in the presence of *BAFF-var* in both conditions (uRBCs and iRBCs), suggesting that CR1 might be translationally upregulated or stabilized in the presence of *BAFF-var* (Figures 7C,D). In contrast to the RNA-seq results, the levels of *MIF* and *ICAM-1* mRNAs did not change (Figures 7A,B); however, MIF and ICAM-1 showed changes in protein expression, indicating altered translation or stability for these proteins. MIF protein levels were slightly but significantly higher in uRBC-WT versus iRBC-WT, while in *BAFF-var* cells, MIF levels were overall higher and did not increase further upon exposure to malaria antigens. This result suggests that the higher levels of sBAFF alone increased MIF expression to a threshold point, which could not be further enhanced by the presence of *P. falciparum* antigens (Figures 7C,D). In contrast to the other genes analyzed, ICAM-1 protein levels were downregulated in B cells purified from *BAFF-var* donors. The significant decrease in the presence of *P. falciparum* antigens suggests that the increased sBAFF level contributes to diminishing ICAM-1 abundance (Figures 7C,D).

B cells expressed higher levels of *NFkB2* mRNA in response to *P. falciparum* antigens in iRBC-var relative to all other groups (uRBC-WT, uRBC-var, and iRBC-WT), suggesting a role for *BAFF-var* in regulating NFKB2 levels in B cells treated with malaria antigens (Figures 7A,B). After activation of the non-canonical NFkB pathway, the p100 (NFKB2) full-length protein is co-translationally processed into the p52 active form that translocates to the nucleus to regulate gene transcription. Western blot analysis revealed that both p100 and p52 were elevated in B cells purified from *BAFF-var* donors. In addition, p52 levels were enhanced in response to malaria antigens in a *BAFF-var*-dependent manner, suggesting that increased p52 protein expression was due to the presence of both *BAFF-var* and *P. falciparum* antigens (Figures 7C,D).

### *BAFF-var* and *P. falciparum* Antigens Modulated mRNA Stability of Select mRNAs

In isolated B cells, the levels of many mRNAs mirrored the levels of the corresponding pre-mRNAs, suggesting that *BAFF-var* and/or *P. falciparum* antigens influenced their levels transcriptionally (e.g., *CXCL10* and *CR1*). However, for others such as *CXCL10* and *NFKB2* mRNAs, changes in the pre-mRNAs levels did not match the changes in steady-state mRNA levels, suggesting that *BAFF-var* and/or *P. falciparum* antigens may not control their transcription, but instead may influence the stability of these mRNAs.

**FIGURE 8:**
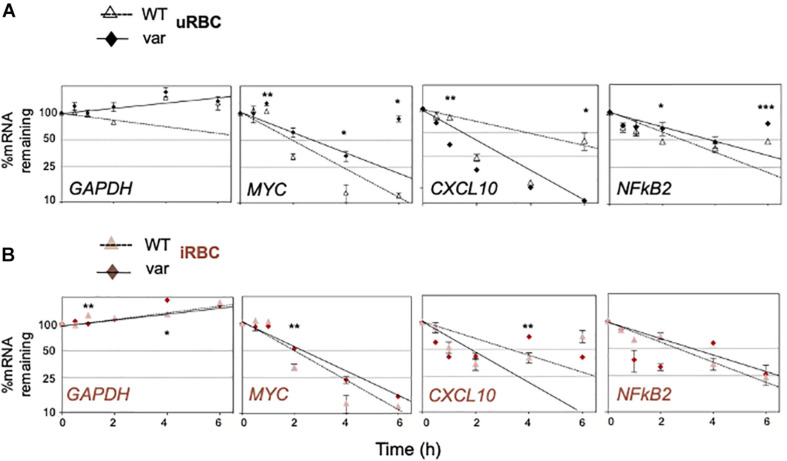
*BAFF-var* genotype and *P. falciparum* antigens regulate the stability of selected mRNAs. PBMCs expressing normal (*BAFF-WT*) or higher (*BAFF-var*) levels of sBAFF were treated for 48 h with lysates from red blood cells uninfected or infected with *P. falciparum* (uRBCs or iRBCs, respectively), and the relative decay rates of target mRNAs were assessed by RT-qPCR analysis after treatment with actinomycin D for the indicated times (0.5, 1, 2, 4, and 6 h). *GAPDH* mRNA, a stable transcript, was included as negative control; *MYC* mRNA was included as a positive unstable control, and mRNA levels were normalized to *18S* rRNA levels. **(A)** Uninfected condition, **(B)** infected condition. Data were plotted on semi-logarithmic scales using Prism. **(A,B)** Data are the means and standard deviation (+SD) from at least three independent experiments. *P < 0.05, **P < 0.01, ***P < 0.005.

To investigate this possibility directly, the half-lives of selected mRNAs were analyzed by treating cells with actinomycin D, which inhibits RNA polymerase II and thus blocks *de novo* transcription. RNA was then collected at different times and assessed by RT-qPCR analysis to calculate the time required to reduce mRNA expression levels to one-half of their initial abundance (*t*_1__/__2_; Figures 8A,B). In the absence of malaria antigens (Figure 8A), we observed a significant loss (*P* < 0.05) of the *CXCL10* mRNA in PBMC-var compared to PBMC-WT. In contrast, *NFKB2* mRNA was more stable in the PBMC-var samples compared to the PBMC-WT cells (Figure 8A). In the presence of *P. falciparum* antigens (Figure 8B), *CXCL10* mRNA was significantly less stable (*P* < 0.05) in PBMC-var than in PBMC-WT, suggesting that a rise in sBAFF reduced *CXCL10* mRNA stability (Figure 8B). The relative stability of *NFKB2* mRNAs in iRBC-treated samples was comparable among all groups (Figure 8B). The expression of *GAPDH* mRNA (a stable transcript) and *MYC* mRNA (an unstable transcript) was used as negative and positive controls, respectively (Figures 8A,B).

### *BAFF-var* Modulates the Production of Cytokines in Response to *P. falciparum* Antigens

**FIGURE 9:**
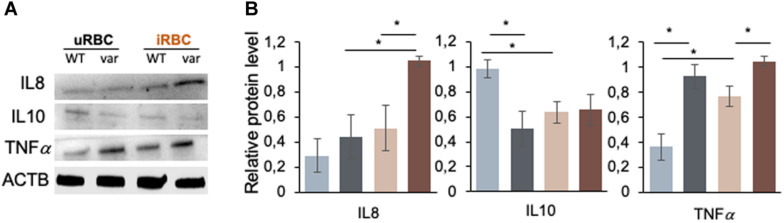
Figure 9 | *BAFF-var* modulates the production of cytokines in response to exposure to *P. falciparum* antigens. Western blot analysis of protein purified from B cells isolated from PBMCs that were treated for 48 h with iRBCs and uRBCs **(A)**. Protein signals were quantified using ImageJ and plotted **(B)**. β-actin (ACTB) served as a loading control. Data in **(B)** are the means and standard deviation (+SD) from at least three independent experiments. *P < 0.05.

We then examined the production of cytokines by B cells after treatment with *P. falciparum* antigens. Specifically, we assessed the levels of cytokines IL8 and IL10, which were found to be differentially produced due to *BAFF-var* status in PBMCs (Figure 3B), and TNFα, which is known to play a key role in the protection and resolution of malaria parasite infection (8) by Western blot analysis of total proteins purified from B cells isolated from PBMCs treated with uRBCs or iRBCs. IL8 production was enhanced in response to malaria antigens in a *BAFF-var*-dependent manner, suggesting that, as observed in PBMCs, increased IL8 protein expression was modulated by the presence of both *BAFF-var* and *P. falciparum* antigens (Figures 9A,B). In contrast, IL10 protein levels significantly decreased in the presence of *P. falciparum* antigens, suggesting that what was observed in total PBMCs was not due to the activation of B cells (Figures 9A,B). Finally, TNFα protein levels changed in the presence of *BAFF-var* in both conditions (uRBCs and iRBCs) (Figures 9A,B). To conclude, IL8 and TNFα production showed a similar trend in B cells and in PBMCs, while IL10 production changed dramatically in B cells as compared with PBMCs.

## Discussion

Here, we sought evidence in support of the hypothesis that *BAFF-var* was positively selected in the Sardinian population due to a protective effect against malaria infection and sought to investigate the underlying mechanisms. To test the hypothesis that *BAFF-var* mediates an enhanced immune response, lysates of erythrocytes infected (iRBCs) or not (uRBCs) with *P. falciparum* were used to treat PBMCs with different BAFF genotype status. The impact of *BAFF-var* on the cellular response to malaria antigens was consistent with a stronger immune response by these cells, including higher levels of sBAFF, B and T cell subsets, immunoglobulins, cytokines, and other molecules implicated in the response to malaria infection.

Here, we found that the feature that most strongly distinguished *BAFF-var* compared with *BAFF-WT* samples after treatment with *P. falciparum* antigens was the expression of β7 integrin on B cell subsets, particularly unswitched memory cells (CD27+ IgD+ CD38− cells). The β7 integrin is an adhesion receptor that dimerizes with the alpha 4 protein and mediates lymphocyte migration and homing to gut-associated lymphoid tissue (GALT) (23). Previous studies have suggested the involvement of mucosal immunity and the upregulation of mucosal specific proteins in malaria infection (17, 18). Nevertheless, the precise function of mucosal immunity in malaria has not yet been elucidated. Here we show for the first time the upregulation of β7 integrin on B cells after stimulation with plasmodium blood-stage parasites, further supporting a possible role of mucosal immunity during malaria infection.

Circulating PD1+ CXCR5+ Th cells are considered similar to follicular helper T cells (Tfh) and are known to play a role in the immune response against *Plasmodium* infection (24). Here, we showed that Tfh2 cells, characterized by the lack of CXCR3 expression, are expanded in *BAFF-var* carriers after stimulation with plasmodium blood-stage parasites. Interestingly, there is evidence that CXCR3-Tfh cells may be superior to CXCR3+ cells in helping B cells to fight *Plasmodium* (24). Indeed, Obeng-Adjei et al. showed that acute plasmodium infection preferentially activates Th1-polarized Tfh cells in children, possibly explaining the relatively short-lived antibody response to natural *P. falciparum* infection in early life. Thus, BAFF-var may contribute to protection against malaria by expanding Tfh subsets that support the development of more durable and protective antibody responses.

The role of immunoglobulins in antimalarial immunity has been analyzed from different points of view in the past years. In early reports, purified IgG antibodies transferred from semi-immune adults to children acutely infected with *P. falciparum* reduced blood-stage parasitemia and disease severity (5). Subsequent studies identified key roles for immunoglobulins in anti-malaria immunity (19, 20, 25). Here, we found a significant variation of IgG1 and IgG3 in the supernatants of *BAFF-var* PBMCs without *P. falciparum* exposure as compared to *BAFF-WT*. No significant changes were observed for IgM. Interestingly, in the presence of *P. falciparum* antigens, the levels of IgG3 and IgM increased in *BAFF-var* PBMCs (Figure 3A). During the immune response to malaria infection, IgG3, along with IgG1, can bind the surface of infected erythrocytes and are the main IgGs able to mediate opsonic phagocytosis of RBCs infected with *P. falciparum*; thus, their increased levels might engender protection against *P. falciparum* infection (26). Furthermore, IgM is recognized as an important functional antibody that targets merozoites and may contribute to naturally acquired protection against malaria (21). Of note, we found a non-significant increase in IgG1 in *BAFF-var* carriers treated with *P. falciparum* antigens.

The cytokine production provides fundamental information regarding the immune responses that can regulate and modify the malaria outcome (27). In our experimental conditions, the presence of *BAFF-var* primarily altered the levels of two key cytokines implicated in the malaria response, IL8 (pro-inflammatory) and IL10 (anti-inflammatory), especially following exposure to *Plasmodium* antigens. The rise in IL8 was more robust than the changes observed for *IL8* mRNA levels, suggesting an increase in translation efficiency of *IL8* in the *BAFF-var* cells (Figure 3B). For all the other cytokines analyzed, differences in expression were only observed in response to *P. falciparum* antigens without any significant effect due to the BAFF genotype (Figure 3B).

To escape the host immune responses, *Plasmodium* parasites may alter the functionality of B cell subsets and immunoglobulin production (27–30). Global analysis of the *BAFF-var* allele on total RNA expression revealed many differentially expressed genes implicated in the immune response to malaria; among them, we selected *CXCL10, CR1, MIF, ICAM-1*, and *NFKB2* (Figure 6) for further analysis. Interestingly, the same genes are also implicated in autoimmune diseases such as systemic lupus erythematosus, rheumatoid arthritis, multiple sclerosis, and Sjogren’s syndrome (31–35). The differential expression of CXCL10, CR1, MIF, ICAM-1, and NFkB2 was validated in B cells from PBMCs and treated with uRBC and iRBC lysates. CXCL10 is a chemokine secreted in response to IFN-γ and involved in chemoattraction of monocytes (36). Exposure to *P. falciparum* antigens elevates *CXCL10* mRNA levels more robustly than *CXCL10* pre-mRNA levels, suggesting a possible increase in *CXCL10* mRNA stability (Figures 8A,B). CXCL10 protein levels further increased in the presence of *BAFF-var*, suggesting a rise in the translational efficiency or protein stability of this cytokine by the variant allele expression (Figures 7C,D). CR1 is a family member of the receptors of the complement activation (RCA) and is expressed on the surface of erythrocytes and phagocytic cells such as macrophages, B cells, neutrophils, and follicular dendritic cells (37). We observed a robust increase in CR1 protein levels in *BAFF-var* cells treated with uRBC and iRBC lysates, but modest changes in *CR1* mRNA levels, suggesting that CR1 translation or protein stability may increase in the presence of *BAFF-var* (Figure 7). Our results support the notion that *BAFF-var* is protective at least in part by inducing CR1, which increases the internalization of immunocomplexes by monocytes/macrophages, decreasing malaria severity (37).

**FIGURE 10:**
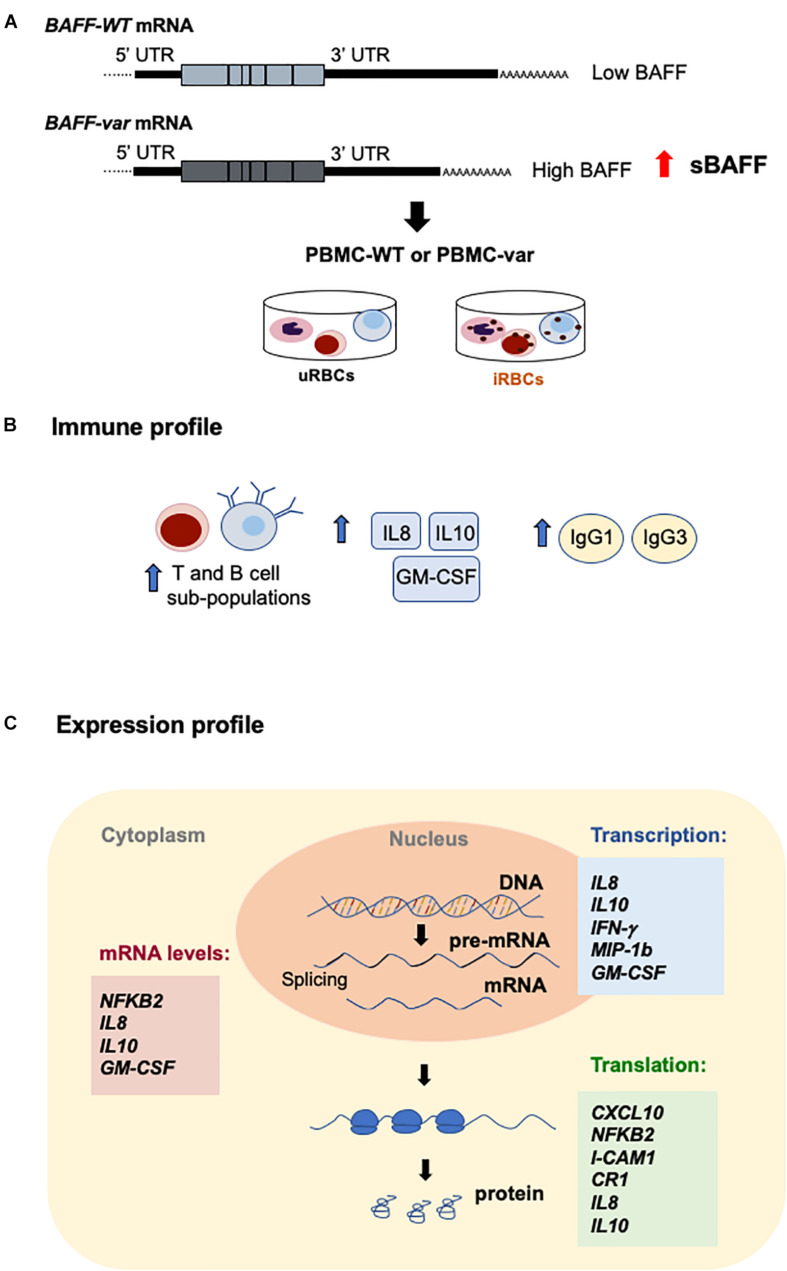
Effects of *BAFF-var* on the response to malaria antigens. **(A)** Schematic representation of the *TNFSF13B*/*BAFF* gene, polyadenylation site (PA), and alternative polyadenylation site (APA). **(B,C)** Proposed model; the presence of *BAFF-var* modulates the subpopulation of T cells and expression of cytokines and immunoglobulins **(B)**; *BAFF-var* also modulates the transcription, mRNA stability, and translation of key genes implicated in the response to *P. falciparum* **(C)**. Collectively, these actions may contribute to mounting a superior immune response to malaria infection.

MIF, macrophage migration inhibitory factor, suppresses IFN-γ and induces IL4 responses during early inflammation, thereby modulating TH1 responses in malaria infection. MIF protein increased significantly by *BAFF-var* in the absence of infection (Figures 7C,D), in agreement with a protective role during *Plasmodium* infection, as elevated MIF levels correlated with the severity of cerebral and placental malaria (38) and with protection from severe childhood malaria (39, 40). ICAM-1 (intercellular adhesion molecule 1), present in different cell types and increased by TNFα treatment, allows the attachment of leukocytes to the endothelium and may permit their subsequent transmigration into peripheral tissue (41). In a model of infection with *Plasmodium berghei ANKA*, ICAM-1-deficient mice survived the acute phase of infection, suggesting that a reduction of ICAM-1 may decrease malaria severity (42). In line with these data, ICAM-1 was reduced in *BAFF-var* cells (Figures 7C,D), suggesting another protective mechanism for *BAFF-var*. Finally, *NFKB2* mRNA, encoding transcription factor NFKB2 (p100), which plays a key role in B cell proliferation and differentiation following the binding of sBAFF to its main receptor, BAFF-R (43), was elevated in our experiments by *P. falciparum* antigens, likely via mRNA stabilization (Figure 8). By contrast, the levels of p52, which complexes with p100 to form functional NFKB2, increased in B cells purified from *BAFF-var* donors treated with malaria antigens through an apparent rise in translation efficiency in the presence of *P. falciparum* antigens (Figures 7C,D). In summary *BAFF-var* drives changes in the production of CXCL10, CR1, MIF, ICAM-1, and NFKB2. These changes are in line with protection against severe malaria asserted by *BAFF-var* (Figure 10).

The pattern of protein expression identified here supports the involvement of *BAFF-var* in controlling immunity and cytokine production in response to malaria antigens. In this regard, BAFF was found to support B cells in numerous ways: survival and maturation, differentiation into antibody secreting cells, and/or immunoglobulin class-switching during malaria infection (16, 44). Furthermore, BAFF drives the expansion of Th1 and Th17 compartments of T cells to increase Th1-associated inflammatory responses (45). In culture, *P. falciparum* antigens enhanced BAFF surface expression and secretion by human monocytes, increasing B cell proliferation and *P. falciparum*-specific IgG expression levels (46). The interaction between BAFF and its receptor BAFF-R activates non-canonical NFκB signaling that is closely associated with the secretion of pro-inflammatory cytokines, chemokines, and adhesion molecules such as ICAM-1, which are important in the malaria immune response (47).

We also analyzed different levels of regulation, both transcriptional and post-transcriptional, but additional studies are needed to validate the specific mechanisms regulating the levels of expression of each protein. In a recent study, we found that NF90 and miR-15a jointly regulated sBAFF production (14). Subsequently, we observed that NF90 regulated the transcription, mRNA stability, and translation of other immune factors implicated in the response to malaria antigens. Specifically, we found that NF90 promoted the production of CCL2, CXCL10, and CR1 in the presence of *P. falciparum* antigens (48). Interestingly, NF90 regulated a few similar proteins identified in the current study, namely, CXCL10 and CR1. Given that NF90 actions are derived from its ability to bind DNA and RNA, and to interact functionally with other RNA-binding proteins (RBPs) and microRNAs, it will be interesting to study which regulators mediate the protective effects of *BAFF-var* and to check whether NF90 influences these gene expression programs studied here.

Overall, our data show that *BAFF-var* modulates the expression of key genes affecting the immune response to *P. falciparum* antigens at different levels. Our results uncover a previously unknown mechanism by which *BAFF-var* increases the circulating sBAFF levels in Sardinian donors, enhancing the expression of numerous mRNAs and proteins, modulating immune cell populations and immunoglobulin representation, thereby potentiating the immune system against *Plasmodium* infection while also increasing the risk of autoimmune diseases such as multiple sclerosis.

## Data Availability Statement

The data has been uploaded to the GEO repository, and assigned GEO accession GSE156102.

## Ethics Statement

The studies involving human participants were reviewed and approved by Sardinian Regional Ethics Committee (prot. n. 2171/CE). The patients/participants provided their written informed consent to participate in this study.

## Author Contributions

VL and MI designed the experiments. VL, MI, and IB performed and analyzed the experiments. VO analyzed the experiments. MF performed the bioinformatic analysis. RM, KA, RG, and MS provided the technical support. PDC provided the expertise and critical feedback. VL, MI, MG, and FC wrote the manuscript. VL, MI, MG, MF, VO, MS, RM, KA, PDC, and FC revised the manuscript. FC and MI designed and directed the project. FC conceived the study. All authors contributed to the article and approved the submitted version.

## Conflict of Interest

The authors declare that the research was conducted in the absence of any commercial or financial relationships that could be construed as a potential conflict of interest.

## Footnotes

1 https://phgkb.cdc.gov

2 https://bioinfogp.cnb.csic.es/tools/venny/
