# Supplementary material for: Evolutionarily Selected Overexpression of the Cytokine BAFF Enhances Mucosal Immune Response Against P. falciparum
Source: Front Immunol. 2020 Oct 6;11:575103. doi: 10.3389/fimmu.2020.575103 (PMC7573158; doi:10.3389/fimmu.2020.575103)
Supplement: Supplementary file 2 [file Image_1.pdf]

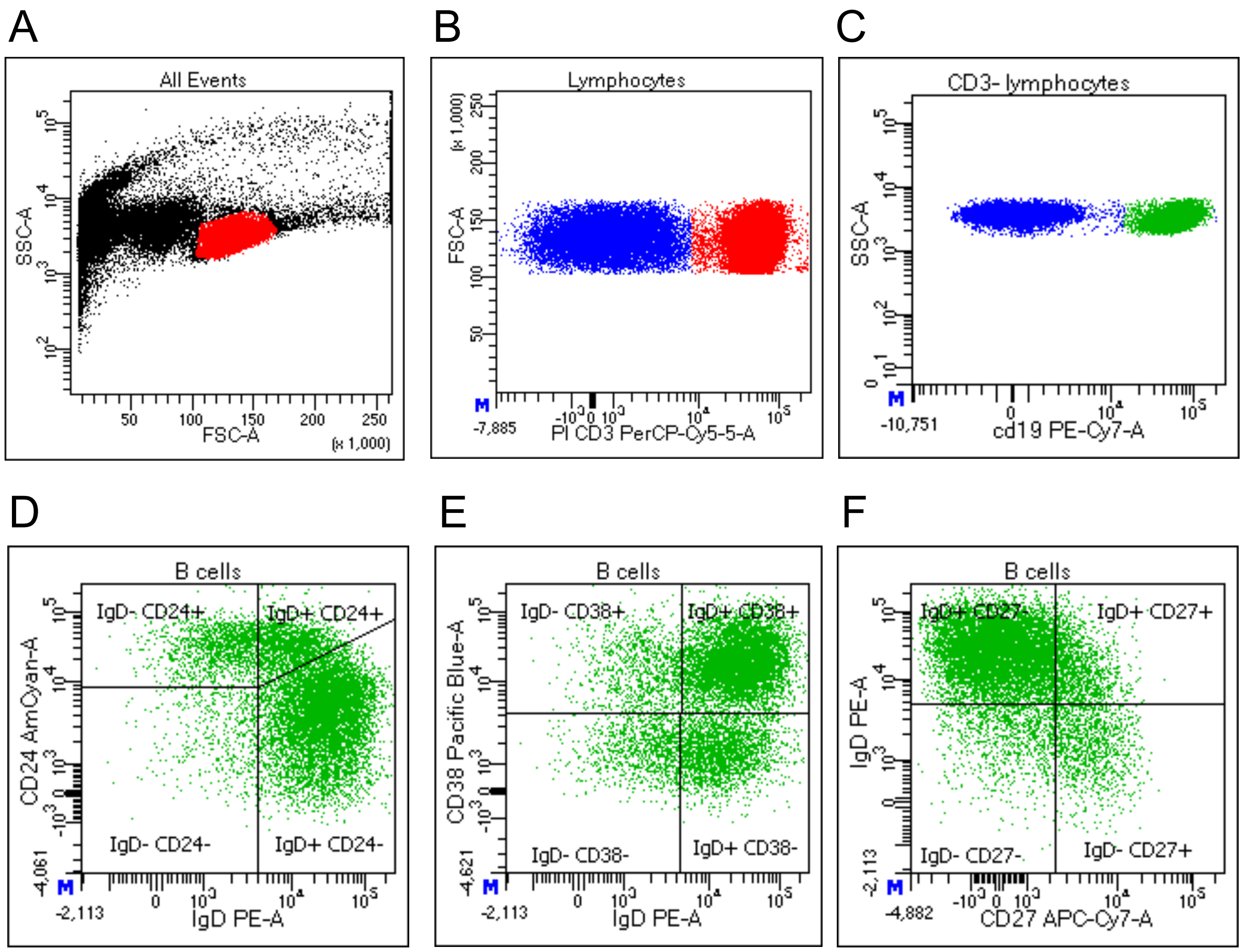

<u>Supplementary Figure S1. Gating strategy for B cells.</u> (A) Lymphocytes (red) were identified based on morphological parameters. (B) Live CD3 negative lymphocytes (blue), which are enriched in B cells, were identified for their simultaneous negativity for propidium iodide (PI) and for CD3 antigen. (C) Within CD3 lymphocytes, CD19 positive cells (green) were considered B cells. (D,E) B cells were divided based on the expression of IgD versus CD38 (D) and IgD versus CD27 markers (E). β7 integrin expression was measured in each B cell subsets (not shown).
