# Supplementary material for: Evolutionarily Selected Overexpression of the Cytokine BAFF Enhances Mucosal Immune Response Against P. falciparum
Source: Front Immunol. 2020 Oct 6;11:575103. doi: 10.3389/fimmu.2020.575103 (PMC7573158; doi:10.3389/fimmu.2020.575103)
Supplement: Supplementary file 3 [file Image_2.pdf]

Supplemental Fig. S2

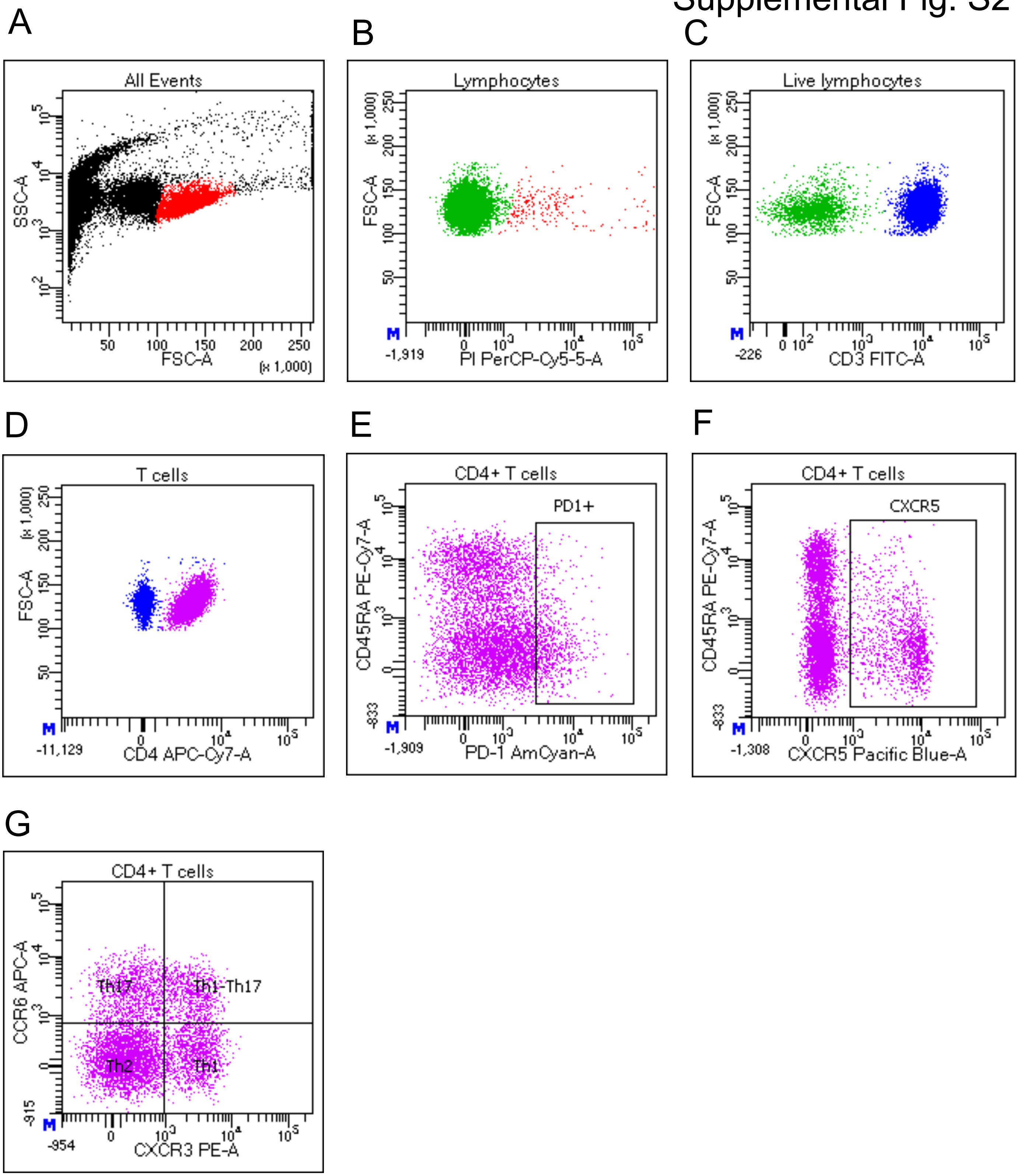

Supplementary Figure S2. Gating strategy for T cells. (A) Lymphocytes (red) were identified based on morphological parameters and (B) live lymphocytes (green) were identified for their negativity for propidium iodide (PI). (C) CD3 positive lymphocytes (blue) were considered T cells. (D) Within T lymphocytes, CD4 positive cells (violet) were considered helper T (Th) cells. (E,F,G) The intersection of PD-1 positive (E) and CXCR5 positive (F) Th cells (by logical gate) were considered follicular helper T cells (Tfh); G) Th cells were divided in Th1, Th2, Th17 and Th1-Th17 based on CXCR3 and CCR6 expression. The intersection of the populations identified in (G) with Tfh cells, by logical gates, allowed the identification of Tfh1, Tfh2, Tfh17 and Tfh1-17.
